# Characteristics Associated With Facebook Use and Interest in Digital Disease Support Among Older Adults With Atrial Fibrillation: Cross-Sectional Analysis of Baseline Data From the Systematic Assessment of Geriatric Elements in Atrial Fibrillation (SAGE-AF) Cohort

**DOI:** 10.2196/15320

**Published:** 2019-11-14

**Authors:** Molly E Waring, Mellanie T Hills, Darleen M Lessard, Jane S Saczynski, Brooke A Libby, Marta M Holovatska, Alok Kapoor, Catarina I Kiefe, David D McManus

**Affiliations:** 1 Department of Allied Health Sciences University of Connecticut Storrs, CT United States; 2 StopAfib.org American Foundation for Women's Health Decatur, TX United States; 3 Department of Population and Quantitative Health Sciences University of Massachusetts Medical School Worcester, MA United States; 4 Department of Pharmacy and Health System Sciences Northeastern University Boston, MA United States; 5 Division of Hospital Medicine Department of Medicine University of Massachusetts Medical School Worcester, MA United States; 6 Division of Cardiology Department of Medicine University of Massachusetts Medical School Worcester, MA United States

**Keywords:** atrial fibrillation, social media, information seeking behavior

## Abstract

**Background:**

Online support groups for atrial fibrillation (AF) and apps to detect and manage AF exist, but the scientific literature does not describe which patients are interested in digital disease support.

**Objective:**

The objective of this study was to describe characteristics associated with Facebook use and interest in digital disease support among older patients with AF who used the internet.

**Methods:**

We used baseline data from the Systematic Assessment of Geriatric Elements in Atrial Fibrillation (SAGE-AF), a prospective cohort of older adults (≥65 years) with AF at high stroke risk. Participants self-reported demographics, clinical characteristics, and Facebook and technology use. Online patients (internet use in the past 4 weeks) were asked whether they would be interested in participating in an online support AF community. Mobile users (owns smartphone and/or tablet) were asked about interest in communicating with their health care team about their AF-related health using a secure app. Logistic regression models identified crude and multivariable predictors of Facebook use and interest in digital disease support.

**Results:**

Online patients (N=816) were aged 74.2 (SD 6.6) years, 47.8% (390/816) were female, and 91.1% (743/816) were non-Hispanic white. Roughly half (52.5%; 428/816) used Facebook. Facebook use was more common among women (adjusted odds ratio [aOR] 2.21, 95% CI 1.66-2.95) and patients with mild to severe depressive symptoms (aOR 1.50, 95% CI 1.08-2.10) and less common among patients aged ≥85 years (aOR 0.27, 95% CI 0.15-0.48). Forty percent (40.4%; 330/816) reported interest in an online AF patient community. Interest in an online AF patient community was more common among online patients with some college/trade school or Bachelors/graduate school (aOR 1.70, 95% CI 1.10-2.61 and aOR 1.82, 95% CI 1.13-2.92, respectively), obesity (aOR 1.65, 95% CI 1.08-2.52), online health information seeking at most weekly or multiple times per week (aOR 1.84, 95% CI 1.32-2.56 and aOR 2.78, 95% CI 1.86-4.16, respectively), and daily Facebook use (aOR 1.76, 95% CI 1.26-2.46). Among mobile users, 51.8% (324/626) reported interest in communicating with their health care team via a mobile app. Interest in app-mediated communication was less likely among women (aOR 0.48, 95% CI 0.34-0.68) and more common among online patients who had completed trade school/some college versus high school/General Educational Development (aOR 1.95, 95% CI 1.17-3.22), sought online health information at most weekly or multiple times per week (aOR 1.86, 95% CI 1.27-2.74 and aOR 2.24, 95% CI 1.39-3.62, respectively), and had health-related apps (aOR 3.92, 95% CI 2.62-5.86).

**Conclusions:**

Among older adults with AF who use the internet, technology use and demographics are associated with interest in digital disease support. Clinics and health care providers may wish to encourage patients to join an existing online support community for AF and explore opportunities for app-mediated patient-provider communication.

## Introduction

Currently, as many as 6 million adults in the United States have atrial fibrillation (AF), and the prevalence of AF is projected to increase to 12 million by 2030 [1]. Both the prevalence and incidence of AF is higher among older adults (ie, aged ≥65 years) compared with younger adults [1], with an estimated prevalence of 1% among adults aged <65 years and 9% among adults aged ≥65 years [2]. Adults with AF are at substantially higher risk of stroke [1], which is 1 of the top 5 leading causes of death in the United States [3].

Treatment with anticoagulants significantly reduces the risk of stroke among adults with AF, but anticoagulants may have significant adverse effects including severe and life-threatening bleeding [4-6] and be difficult to manage (eg, necessity for regular monitoring, dosing changes, and dietary restrictions) [7]. While education and behavioral interventions may improve adherence and persistence with treatment, a recent systematic review did not find that interventions consisting of self-monitoring plus education increased time in therapeutic range compared with usual care [8]. Digital health approaches may be an effective strategy for helping adults with AF manage their disease [9], and pilot studies appear promising [10,11].

Although fewer older US adults aged ≥65 years go online, own mobile devices, and use social media compared with younger adults, technology adoption among older US adults has nearly quadrupled since 2000 [12]. Currently, two-thirds of older US adults are online, 42% own a smartphone, 32% own a tablet computer, and 34% use social media [12]. Previous research indicates that there is interest among older adults with cardiovascular disease to communicate with their health care teams via social media and that greater use of Facebook may be a predictor of greater willingness to participate in online patient support communities [13]. Online support groups for AF exist, and recent research suggests that patients participating in these communities benefit from connecting with others with AF for information and support related to managing their health, including information and support related to the risks and benefits of treatment options, personal experiences, and medication management [14,15].

However, existing literature does not illuminate the characteristics of older adults with AF interested in joining an online support community for AF. Similarly, apps for the detection and management of AF are being developed [16-21], but, similarly, previous research has not examined which older adults with AF would be interested in utilizing this technology to communicate with their health care team. The purpose of this study was to describe, in a cohort of older patients with AF who used the internet, patient characteristics associated with the use of social media and interest in digital disease support. Specifically, we examined the extent to which demographic, clinical, and lifestyle characteristics were associated with (1) Facebook use, (2) interest in an online AF patient support community among older patients with AF, and (3) interest in using a mobile app to communicate with their health care team.

## Methods

### Study Design and Data Collection

We used data from the Systematic Assessment of Geriatric Elements in Atrial Fibrillation (SAGE-AF) study. Between 2016 and 2018, SAGE-AF enrolled 1244 older adults with AF at high stroke risk from 7 clinical sites in central and eastern Massachusetts or central Georgia. Staff prescreened patients scheduled to attend a clinic visit and sent eligible patients an invitation to participate in the study 1 week before their appointment. Eligibility criteria for SAGE-AF included having a scheduled ambulatory care visit at one of the study practices, electrocardiographic evidence of AF, being aged ≥65 years, and having a CHA_2_DS_2_VASC risk score ≥2. Exclusion criteria were documentation of an absolute contraindication to oral anticoagulants (eg, recent major bleeding), indication for oral anticoagulants other than AF (eg, venous thromboembolism), inability to demonstrate capacity to provide informed consent as assessed by a capacity instrument that combines direct questions about their understanding of study participation with interviewer observations of the patient [22], not English speaking, planned invasive procedure with high risk for uncontrollable bleeding, current pregnancy, prisoner status, and unwillingness or inability to participate in planned 1- and 2-year follow-up visits at their study sites. Data were collected through a comprehensive geriatric assessment, structured interviews, and abstraction of electronic medical records. Data for this study were derived from the baseline assessment. All participants provided written informed consent. SAGE-AF was approved by the institutional review boards at each study site. Participants received a US $60 gift card after completing the 60-min baseline assessment.

### Measures

The baseline interview included questions about the use of technology and social media adapted from the Pew Research Center [12,23] and interest in digital disease support developed in previous research [24]. Participants reported whether they had gone online or accessed the internet over the past 4 weeks (response options: not at all in the past 4 weeks, less than once a week, once a week, more than once a week but not every day, once a day, or more than once a day). We defined online patients as patients who reported using the internet at least once during the past 4 weeks. Online patients were asked whether they had a Facebook account. Online patients were also asked the following:

If we were to create an online community (via a private website or an app) specifically designed for patients with atrial fibrillation, how interested would you be in participating? The community would be held through a private website and/or a secure smartphone/tablet app. You could use this community to ask questions about afib, set activity or diet goals, or report progress on a regular basis.

We combined no and unsure responses (vs yes) to highlight participants expressing clear interest. Participants were asked if they owned a smartphone (eg, iPhone, Android phone, Windows phone, or Blackberry) or tablet computer (eg, iPad, Samsung Galaxy, Motorola Xoom, or Kindle Fire). Participants who reported owning a smartphone and/or tablet computer were categorized as mobile users. Mobile users were asked “would you be interested in communicating with your doctor or health care team about your atrial fibrillation-related health using a secure smartphone or tablet app?” We combined no and unsure responses (vs yes) to highlight participants expressing clear interest.

Participants self-reported demographics including race/ethnicity, education level, marital status, and living situation during the baseline interview. We abstracted age, height, weight, and medical history variables from patients’ medical records at baseline, including comorbidities (eg, type II diabetes, hypertension, stroke, heart failure, and cancer), whether the patient had newly diagnosed or prevalent AF, use of anticoagulants, and whether the patient’s AF was managed by a dedicated anticoagulation clinic. We calculated body mass index (BMI) from height and weight abstracted from medical records and categorized participants’ weight status as underweight (BMI<18.5 kg/m^2^), normal weight (18.5 kg/m^2^≤BMI<25 kg/m^2^), overweight (25 kg/m^2^≤BMI<30 kg/m^2^), or obese (30 kg/m^2^≤BMI) [25].

Participants were asked “how much difficulty do you have reading ordinary print in newspapers?” and “how much difficulty do you have doing work or hobbies that require you to see well up close, such as cooking, sewing, fixing things around the house, or using hand tools?” (response options: no difficulty at all, a little difficulty, moderate difficulty, extreme difficulty, stopped doing this because of your eyesight, stopped doing this because of other reasons, or no interest in doing this). Participants who reported moderate or extreme difficulty or reported stopping activity because of eyesight for either question were considered to have moderate/extreme/activity-limiting difficulty with reading text. Depressive symptoms were assessed using the Patient Health Questionnaire-9 [26]. This 9-item questionnaire asks participants to self-report the frequency with which they have experienced depressive symptoms over the past 2 weeks (response options: not at all, several days, more than half the days, or nearly every day). We calculated a total score from the sum of responses, with a potential range of 0 to 27 [26]. As few participants reported depressive symptoms in the moderate to severe range, we dichotomized symptoms as minimal (0-4) versus mild or more severe symptomology (≥5) [26]. Symptoms of anxiety were assessed using the Generalized Anxiety Disorder-7 measure [27]. This 7-item scale asks participants to self-report the frequency with which they have experienced symptoms of anxiety over the past 2 weeks (response options: not at all, several days, over half the days, or nearly every day). We summed scores to generate a total score representing symptoms of anxiety, with a potential range of 0 to 21 [27]. As few participants reported symptoms of anxiety in the moderate to severe range, we dichotomized symptoms as minimal (0-4) versus mild or more severe symptomology (≥5+) [27]. The Perceived Efficacy in Patient-Physician Interactions is a 10-item validated, reliable measure of self-efficacy in patient-physician interactions [28], with total scores ranging from 5 to 50 [29]. We categorized scores of ≥45 as high perceived efficacy in patient-provider interactions; this score is equivalent to average responses of very or extremely confident.

Participants were asked to report how much they were bothered by AF based on experiencing heart palpitations (ie, hear fluttering, skipping, or racing), irregular heartbeat (feeling any pause in heart activity), lightheadedness, or dizziness (response options: not at all bothered or I did not have this symptom, hardly bothered, a little bothered, moderately bothered, quite a bit bothered, very bothered, or extremely bothered). We categorized participants as being quite/very/extremely bothered by 1 or more of these 4 symptoms over the past 4 weeks. Participants were asked how satisfied they were with how well their current treatment controlled their AF; responses were categorized as very/extremely satisfied, somewhat satisfied, or mixed satisfied and dissatisfied or somewhat/very/extremely dissatisfied. Participants were asked “in the past month, how much help with the management of your atrial fibrillation have you needed?” (response options: none, very little, some, quite a bit, or very much); responses were dichotomized as none versus any needed assistance.

Participants with Facebook accounts were asked how often they checked their accounts over the past 4 weeks (response options: not at all in the past 4 weeks, less than once a week, once a week, more than once a week but not every day, once a day, or more than once a day); we collapsed response options to not at all, less than once a week, weekly, and daily. Online participants (ie, those who reporting using the internet in the past 4 weeks) were asked how often they used the internet to look for advice or information about their health (response options: not at all in the past 4 weeks, less than once a week, once a week, more than once a week but not every day, once a day, or more than once a day). Online health information seeking was collapsed as not at all, at most weekly, or multiple times per week. Mobile users were asked whether they had any apps related to their health (yes vs no/unsure).

### Statistical Analysis

Only online patients (ie, patients who reported using the internet) were asked about the use of Facebook and interest in an online support community for AF. Therefore, these analyses were limited to online patients (ie, patients who reported internet use). Only patients who reported owning a tablet computer and/or smartphone were asked about their interest in using a mobile app to communicate with their health care team. Therefore, analyses examining interest in app-mediated communication were limited to mobile users (ie, patients who have tablet computers and/or smartphones). We additionally excluded participants missing any of the characteristics examined.

We compared demographic characteristics of SAGE-AF participants excluded with characteristics of participants included in the analytic sample using *t* tests for age and chi-squared tests for gender and race/ethnicity. We used logistic regression models to identify crude and multivariable predictors of Facebook use and interest in digital disease support. As marital status and living situation were highly related (only 3 patients who were married or living as married reported living alone), we considered living situation for inclusion in regression models and describe marital status of participants but did not consider this variable for inclusion in regression models. To identify multivariable predictors, we included variables that were associated with the outcome at *P*<.10 and retained variables in the model if the odds ratio (OR) was statistically significant at the .05 level for any level of the variable. We additionally considered study site (Massachusetts vs Georgia) for inclusion in adjusted models. However, as study site was not statistically significant in any of the 3 models and estimated ORs for participant characteristics were very similar to models that did not include study site (data not shown), the final adjusted models did not include study site. Analyses were conducted using SAS 9.4 (SAS Inc, Cary, NC).

## Results

### Characteristics of the Sample

Seventy percent (875/1244) of the patients enrolled in the SAGE-AF cohort reported using the internet in the previous 4 weeks (online patients). We excluded online patients who lived in a nursing home (n=4) those missing information about Facebook use (n=3), those missing information about interest in an online AF patient community (n=1), those missing information about interest in using a mobile app to communicate with their health care team (n=4), and patients missing information on any of the characteristics examined (n=47), resulting in an analytic sample of 816 online older adults with AF. SAGE-AF participants excluded from the analytic sample were on average 3.7 years older than participants in analytic sample (mean 78.0, SD 7.4 years vs mean 74.2, SD 6.6 years; *P*<.001) and less likely to be non-Hispanic white (73.1% vs 91.1%; *P*<.001); excluded and included participants were similarly likely to be female (50.7% vs 47.8%; *P*=.33).

Online patients were on average aged 74.2 (SD 6.6) years, 47.8% were female, and 91.1% were non-Hispanic white. Almost all (98.9%) had prevalent AF at enrollment. Six out of 10 participants reported seeking health information online; 19.6% of the sample looked online for health information more than once a week during the past 4 weeks, 39.3% at most once per week, and 41.1% not at all. Among mobile users, 29.6% reported using health-related mobile apps. Additional demographic, clinical, and psychosocial characteristics are shown in Table 1.

### Characteristics Associated With Facebook Use

Just over half (52.5%) of online patients reported using Facebook. Among Facebook users, 16.4% reported using Facebook less than once a week, 24.3% weekly, and 59.4% daily. Facebook use was more common among women than men (62.6% vs 43.2%; adjusted OR [aOR] 2.21, 95% CI 1.66-2.95) and among patients with mild to severe depressive symptoms (61.2% vs 49.3%; aOR 1.50, 95% CI 1.08-2.10) and less common among the oldest patients (31.9% vs 60.3%; aOR 0.27, 95% CI 0.15-0.48 for patients aged ≥85 years compared with patients aged 65 to 69 years; Table 2).

**Table 1:** Demographic, clinical, and psychosocial characteristics of older adults with atrial fibrillation (AF) who used the internet (N=816), Systematic Assessment of Geriatric Elements in Atrial Fibrillation (SAGE-AF) 2016-2018.

Participant characteristics		Value, n (%)
**Age (years)**		
	65-69	224 (27.5)
	70-74	254 (31.1)
	75-84	266 (32.6)
	≥85	72 (8.8)
Female		390 (47.8)
Non-Hispanic white		743 (91.1)
**Marital status**		
	Married or living as married	504 (61.9)
	Divorced or separated	109 (13.4)
	Widowed	162 (19.9)
	Single	39 (4.8)
Lives alone		213 (26.1)
**Education**		
	High school/General Educational Development or less	177 (21.7)
	Some college or trade school	215 (26.4)
	College/some graduate coursework	143 (17.5)
	Graduate degree	281 (34.4)
**Body mass index**		
	Underweight	6 (0.7)
	Normal weight	141 (17.3)
	Overweight	279 (34.2)
	Obese	390 (47.8)
History of type II diabetes		197 (24.1)
History of myocardial infarction		145 (17.8)
History of cancer		253 (31.0)
Moderate/extreme/activity-limiting difficulty reading text (eg, newspaper)		119 (14.6)
Elevated depressive symptoms		214 (26.2)
Elevated anxiety symptoms		178 (21.8)
High perceived efficacy in patient-provider interactions		544 (66.7)
Quite/very/extremely bothered by ≥1 of 4 AF symptoms in the past 4 weeks		92 (11.3)
**Satisfaction with current AF treatment**		
	Very/extremely satisfied	637 (78.1)
	Somewhat satisfied	97 (11.9)
	Mixed satisfied and dissatisfied, or somewhat, very, or extremely dissatisfied	82 (10.1)
Needed help managing AF in the past 4 weeks		118 (14.5)
**Anticoagulant management**		
	Not taking anticoagulant	432 (52.9)
	On anticoagulant, managed by anticoagulation clinic	259 (31.7)
	On anticoagulant, not managed by anticoagulation clinic	125 (15.3)

**Table 2:** Use of Facebook in relation to demographic, clinical, psychosocial, and technology use characteristics of online older adults with atrial fibrillation (N=816), Systematic Assessment of Geriatric Elements in Atrial Fibrillation (SAGE-AF) 2016-2018.

Participant characteristics		Uses Facebook		
		Value, n (%)	Crude OR^a^ (95% CI)	Adjusted OR (95% CI)
**Age (years)**				
	65-69	135 (60.3)	Reference	Reference
	70-74	133 (52.4)	0.73 (0.50-1.04)	0.71 (0.49-1.04)
	75-84	137 (51.5)	0.70 (0.49-1.00)	0.67 (0.46-0.97)
	≥85	23 (32)	0.31 (0.18-0.54)	0.27 (0.15-0.48)
**Sex**				
	Male	184 (43.2)	Reference	Reference
	Female	244 (62.6)	2.20 (1.67-2.91)	2.21 (1.66-2.95)
**Race/ethnicity**				
	Non-Hispanic white	386 (52.0)	Reference	—^b^
	Other race/ethnicity	42 (58)	1.25 (0.77-2.04)	—
**Living situation**				
	Lives with others	319 (52.9)	Reference	—
	Lives alone	109 (51.2)	0.93 (0.68-1.28)	—
**Education**				
	High school/General Educational Development or less	100 (56.5)	Reference	—
	Some college or trade school	124 (57.7)	1.05 (0.70-1.57)	—
	College/graduate coursework	68 (47.6)	0.70 (0.45-1.09)	—
	Graduate degree	136 (48.4)	0.72 (0.50-1.05)	—
**Body mass index**				
	Underweight	4 (66.7)	2.62 (0.47-14.79)	—
	Normal weight	61 (43.3)	Reference	—
	Overweight	145 (52.0)	1.42 (0.94-2.13)	—
	Obese	218 (55.9)	1.66 (1.13-2.45)	—
**History of type II diabetes**				
	No	319 (51.5)	Reference	—
	Yes	109 (55.3)	1.17 (0.84-1.61)	—
**History of myocardial infarction**				
	No	349 (52.0)	Reference	—
	Yes	79 (54.5)	1.10 (0.77-1.58)	—
**History of cancer**				
	No	295 (52.4)	Reference	—
	Yes	133 (52.6)	1.01 (0.75-1.36)	—
**Difficulty reading text (eg, newspaper)**				
	Not difficult at all/a little difficult	368 (52.8)	Reference	—
	Moderate/extreme/activity-limiting difficulty	60 (50.4)	0.91 (0.62-1.34)	—
**Depressive symptoms**				
	Minimal symptoms (0-4)	297 (49.3)	Reference	Reference
	Mild to severe symptoms (5+)	131 (61.2)	1.62 (1.18-2.23)	1.50 (1.08-2.10)
**Anxiety symptoms**				
	Minimal symptoms (0-4)	327 (51.3)	Reference	—
	Mild to severe symptoms (5+)	101 (56.7)	1.25 (0.89-1.74)	—
**High perceived efficacy in patient-provider interactions**				
	Less confident (<45)	143 (52.6)	Reference	—
	Very/extremely confident (45+)	285 (52.4)	0.99 (0.74-1.33)	—
**How bothered by 4 AF** ^c^ **symptoms in the past 4 weeks**				
	At most moderately bothered by any symptoms	370 (51.1)	Reference	—
	Quite/very/extremely bothered by ≥1 symptom	58 (63)	1.63 (1.04-2.55)	—
**Satisfaction with current AF treatment**				
	Very/extremely satisfied	322 (50.6)	Reference	—
	Somewhat satisfied	62 (64)	1.73 (1.11-2.70)	—
	Mixed satisfied and dissatisfied, or somewhat, very, or extremely dissatisfied	44 (54)	1.13 (0.71-1.80)	—
**Needed help managing AF in the past 4 weeks**				
	None	369 (52.9)	Reference	—
	Very little/some/quite a lot/very much	59 (50.0)	0.89 (0.60-1.32)	—
**Anticoagulant management**				
	Not taking anticoagulant	231 (53.5)	Reference	—
	On anticoagulant, managed by anticoagulation clinic	132 (51.0)	0.90 (0.66-1.23)	—
	On anticoagulant, not managed by anticoagulation clinic	65 (52.0)	0.94 (0.63-1.40)	—
**Online health information seeking in the past 4 weeks**				
	Not at all	165 (49.3)	Reference	—
	At most once a week	174 (54.2)	1.22 (0.90-1.66)	—
	Multiple times per week	89 (55.6)	1.29 (0.89-1.89)	—

^a^OR: odds ratio.

^b^Not included in the adjusted regression model.

^c^AF: atrial fibrillation.

### Characteristics Associated With Interest in an Online Atrial Fibrillation Patient Community

Forty percent (40.4%) of online patients reported interest in an online AF patient community. Patients with some postsecondary education (some college or trade school) and those with a bachelor’s degree or some graduate education were more likely to report interest in an online AF patient community than patients with a high school education or less (45.1% and 49.0% vs 32.2%; aOR 1.70, 95% CI 1.10-2.61 and aOR 1.82, 95% CI 1.13-2.92, respectively; Table 3). Patients with obesity were more likely to report interest in an online AF patient community than patients who were normal weight (45.4% vs 31.9%; aOR 1.65, 95% CI 1.08-2.52; Table 3). More frequent online health information seeking was associated with greater likelihood of expressing interest in an online AF patient community (55.6% and 43.9% vs 29.9%; aOR 1.84, 95% CI 1.32-2.56 for at most weekly online health information seeking and aOR 2.78, 95% CI 1.86-4.16 for online health information seeking multiple times weekly; Table 3). Finally, online patients who used Facebook daily were more likely to express interest in an online AF patient community than patients who did not use Facebook (50.0% vs 34.8%; aOR 1.76, 95% CI 1.26-2.46; Table 3).

**Table 3:** Interest in online atrial fibrillation patient community in relation to demographic, clinical, psychosocial, and technology use characteristics of online older adults with atrial fibrillation (N=816), Systematic Assessment of Geriatric Elements in Atrial Fibrillation (SAGE-AF) 2016-2018.

Participant characteristics		Interest in an online AF^a^ patient community		
		Value, n (%)	Crude OR^b^ (95% CI)	Adjusted OR (95% CI)
**Age (years)**				
	65-69	101 (45.1)	Reference	—^c^
	70-74	113 (44.5)	0.98 (0.68-1.40)	—
	75-84	98 (36.8)	0.71 (0.49-1.02)	—
	≥85	18 (25)	0.41 (0.22-0.74)	—
**Sex**				
	Male	175 (41.1)	Reference	—
	Female	155 (39.7)	0.95 (0.72-1.25)	—
**Race/ethnicity**				
	Non-Hispanic white	302 (40.7)	Reference	—
	Other race/ethnicity	28 (38)	0.91 (0.55-1.49)	—
**Living situation**				
	Lives with others	250 (41.5)	Reference	—
	Lives alone	80 (37.6)	0.85 (0.62-1.17)	—
**Education**				
	High school/General Educational Development or less	57 (32.2)	Reference	Reference
	Some college or trade school	97 (45.1)	1.73 (1.14-2.62)	1.70 (1.10-2.61)
	College/graduate school	70 (49.0)	2.02 (1.28-3.18)	1.82 (1.13-2.92)
	Graduate degree	106 (37.7)	1.28 (0.86-1.90)	1.19 (0.78-1.81)
**Body mass index**				
	Underweight	3 (50.0)	2.13 (0.41-10.99)	2.29 (0.43-12.14)
	Normal weight	45 (31.9)	Reference	Reference
	Overweight	105 (37.6)	1.29 (0.84-1.98)	1.25 (0.80-1.94)
	Obese	177 (45.4)	1.77 (1.18-2.66)	1.65 (1.08-2.52)
**History of type II diabetes**				
	No	246 (39.7)	Reference	—
	Yes	84 (42.6)	1.13 (0.81-1.56)	—
**History of myocardial infarction**				
	No	268 (39.9)	Reference	—
	Yes	62 (42.8)	1.12 (0.78-1.62)	—
**History of cancer**				
	No	230 (40.9)	Reference	—
	Yes	100 (39.5)	0.95 (0.70-1.28)	—
**Difficulty reading text (eg, newspaper)**				
	Not difficult at all/a little difficult	290 (41.6)	Reference	—
	Moderate/extreme/activity-limiting difficulty	40 (33.6)	0.71 (0.47-1.07)	—
**Depressive symptoms**				
	Minimal symptoms (0-4)	232 (38.5)	Reference	—
	Mild to severe symptoms (5+)	98 (45.8)	1.35 (0.98-1.85)	—
**Anxiety symptoms**				
	Minimal symptoms (0-4)	241 (37.8)	Reference	—
	Mild to severe symptoms (5+)	89 (50.0)	1.65 (1.18-2.30)	—
**High perceived efficacy in patient-provider interactions**				
	Less confident (<45)	108 (39.7)	Reference	—
	Very/extremely confident (45+)	222 (40.8)	1.05 (0.78-1.41)	—
**How bothered by AF symptoms in the past 4 weeks**				
	At most moderately bothered by any symptom	281 (38.8)	Reference	—
	Quite/very/extremely bothered by ≥1 symptom	49 (53)	1.80 (1.16-2.78)	—
**Satisfaction with current AF treatment**				
	Very/extremely satisfied	241 (37.8)	Reference	—
	Somewhat satisfied	45 (46)	1.42 (0.93-2.19)	—
	Mixed satisfied and dissatisfied, or somewhat, very, or extremely dissatisfied	44 (54)	1.90 (1.20-3.02)	—
**Needed help managing AF in the past 4 weeks**				
	None	280 (40.1)	Reference	—
	Very little/some/quite a lot/very much	50 (42.4)	1.10 (0.74-1.63)	—
**Anticoagulant management**				
	Not taking anticoagulant	181 (41.9)	Reference	—
	On anticoagulant, managed by AC^d^ clinic	104 (40.2)	0.93 (0.68-1.27)	—
	On anticoagulant, not managed by AC clinic	45 (36.0)	0.78 (0.52-1.18)	—
**Online health information seeking in the past 4 weeks**				
	Not at all	100 (29.9)	Reference	Reference
	At most once a week	141 (43.9)	1.84 (1.34-2.54)	1.84 (1.32-2.56)
	Multiple times per week	89 (55.6)	2.95 (1.99-4.35)	2.78 (1.86-4.16)
**Frequency of Facebook use in the past 4 weeks**				
	Does not use Facebook	135 (34.8)	Reference	Reference
	Less than once a week over the past 4 weeks	24 (34)	0.98 (0.57-1.67)	0.96 (0.55-1.66)
	Weekly over the past 4 weeks	44 (42.3)	1.37 (0.88-2.14)	1.32 (0.84-2.08)
	Daily over the past 4 weeks	127 (50.0)	1.87 (1.36-2.59)	1.76 (1.26-2.46)

^a^AF: atrial fibrillation.

^b^OR: odds ratio.

^c^Not included in the adjusted regression model.

^d^AC: anticoagulation.

### Characteristics Associated With Interest in Using a Mobile App to Communicate With Their Health Care Team Among Mobile Users

A total of 60.2% of online patients reported owning a tablet computer and 58.2% owned a smartphone; 76.7% were mobile users. Among mobile users, 51.8% reported interest in using a mobile app to communicate with their health care team. Women were less likely to express interest in using mobile apps to communicate with their health care team (42.7% vs 60.6%; aOR 0.48, 95% CI 0.34-0.68). Interest in app-mediated communication was more common among individuals who had completed trade school/some college versus high school/General Educational Development (54.4% vs 36.0%; aOR 1.95, 95% CI 1.17-3.22; Table 4). More frequent online health information seeking was associated with greater likelihood of expressing interest in app-mediated communication with their health care team (57.6% and 64.4% vs 38.0%; aOR 1.86, 95% CI 1.27-2.74 for at most weekly online health information seeking and aOR 2.24, 95% CI 1.39-3.62 for online health information seeking multiple times weekly; Table 4). Patients who have health-related apps were more likely to report interest in communicating with their health care team via a mobile app (75.7% vs 41.7%; aOR 3.92, 95% CI 2.62-5.86; Table 4).

**Table 4:** Interest in using mobile app to communicate with health care team in relation to demographic, clinical, psychosocial, and technology use characteristics of online older adults with atrial fibrillation who owned mobile devices (n=626), Systematic Assessment of Geriatric Elements in Atrial Fibrillation (SAGE-AF) 2016-2018.

Participant characteristics		Interest in using mobile app to communicate with health care team		
		Value, n (%)	Crude OR^a^ (95% CI)	Adjusted OR (95% CI)
**Age (years)**				
	65-69	114 (58.8)	Reference	—^b^
	70-74	107 (55.7)	0.88 (0.59-1.32)	—
	75-84	90 (46.2)	0.60 (0.40-0.90)	—
	≥85	13 (29)	0.29 (0.14-0.58)	—
**Sex**				
	Male	192 (60.6)	Reference	Reference
	Female	132 (42.7)	0.49 (0.35-0.67)	0.48 (0.34-0.68)
**Race/ethnicity**				
	Non-Hispanic White	292 (51.3)	Reference	—
	Other race/ethnicity	32 (56)	1.21 (0.70-2.10)	—
**Living situation**				
	Lives with others	254 (54.0)	Reference	—
	Lives alone	70 (44.9)	0.69 (0.48-1.00)	—
**Education**				
	High school/General Educational Development or less	46 (36.0)	Reference	Reference
	Some college or trade school	92 (54.4)	2.10 (1.31-3.37)	1.95 (1.17-3.22)
	College/graduate school	64 (56.1)	2.25 (1.34-3.78)	1.64 (0.94-2.87)
	Graduate degree	122 (56.5)	2.29 (1.46-3.59)	1.58 (0.97-2.58)
**Body mass index**				
	Underweight	1 (33.3)	0.63 (0.06-7.16)	—
	Normal weight	43 (44.3)	Reference	—
	Overweight	119 (55.9)	1.59 (0.98-2.58)	—
	Obese	161 (51.4)	1.33 (0.84-2.10)	—
**History of type II diabetes**				
	No	242 (51.0)	Reference	—
	Yes	82 (54.3)	1.14 (0.79-1.65)	—
**History of myocardial infarction**				
	No	270 (52.4)	Reference	—
	Yes	54 (48.7)	0.86 (0.57-1.30)	—
**History of cancer**				
	No	226 (52.8)	Reference	—
	Yes	98 (49.5)	0.88 (0.63-1.23)	—
**Difficulty reading text (eg, newspaper)**				
	Not difficult at all/a little difficult	277 (51.2)	Reference	—
	Moderate/extreme/activity-limiting difficulty	47 (55)	1.18 (0.74-1.87)	—
**Depressive symptoms**				
	Minimal symptoms (0-4)	239 (51.5)	Reference	—
	Mild to severe symptoms (5+)	85 (52.5)	1.04 (0.73-1.49)	—
**Anxiety symptoms**				
	Minimal symptoms (0-4)	249 (51.6)	Reference	—
	Mild to severe symptoms (5+)	75 (52.5)	1.04 (0.71-1.51)	—
**High perceived efficacy in patient-provider interactions**				
	Less confident (<45)	104 (51.2)	Reference	—
	Very/extremely confident (45+)	220 (52.0)	1.03 (0.74-1.44)	—
**How bothered by AF^c^ symptoms in the past 4 weeks**				
	At most moderately bothered by any symptom	286 (52.1)	Reference	—
	Quite/very/extremely bothered by ≥1 symptom	38 (49)	0.90 (0.56-1.44)	—
**Satisfaction with current AF treatment**				
	Very/extremely satisfied	249 (51.0)	Reference	—
	Somewhat satisfied	38 (50)	0.96 (0.59-1.56)	—
	Mixed satisfied and dissatisfied or somewhat, very, or extremely dissatisfied	37 (60)	1.42 (0.83-2.43)	—
**Needed help managing AF in the past 4 weeks**				
	None	274 (51.3)	Reference	—
	Very little/some/quite a lot/very much	50 (54)	1.13 (0.72-1.76)	—
**Anticoagulant management**				
	Not taking anticoagulant	181 (52.6)	Reference	—
	On anticoagulant, managed by anticoagulation clinic	110 (55.6)	1.13 (0.79-1.60)	—
	On anticoagulant, not managed by anticoagulation clinic	33 (39)	0.58 (0.36-0.95)	—
**Online health information seeking in the past 4 weeks**				
	Not at all	89 (38.0)	Reference	Reference
	At most once a week	148 (57.6)	2.21 (1.54-3.18)	1.86 (1.27-2.74)
	Multiple times per week	87 (64.4)	2.95 (1.90-4.59)	2.24 (1.39-3.62)
**Frequency of Facebook use in the past 4 weeks**				
	Does not use Facebook	126 (47.4)	Reference	—
	Less than once a week over the past 4 weeks	28 (57)	1.48 (0.80-2.74)	—
	Weekly over the past 4 weeks	41 (48)	1.04 (0.64-1.69)	—
	Daily over the past 4 weeks	129 (57.1)	1.48 (1.03-2.11)	—
**Has apps related to health**				
	No/unsure	184 (41.7)	Reference	Reference
	Yes	140 (75.7)	4.35 (2.96-6.39)	3.92 (2.62-5.86)

^a^OR: odds ratio.

^b^Not included in the adjusted regression model.

^c^AF: atrial fibrillation.

## Discussion

### Principal Findings

In this contemporary community-based cohort of older patients with AF, we found that 70% used the internet and three-quarters were mobile users (ie, owned a smartphone or tablet computer). Among online patients, just over half used Facebook and 40% were interested in an online community for patients with AF. Among mobile users, 52% were interested in using a mobile app to communicate with their health care team. Women, younger patients, and those with elevated depressive symptoms were more likely to use Facebook. More educated patients, patients with obesity, frequent Facebook users, and those engaging in digital activities related to health were more likely to express interest in digital disease support. Men were also more likely to report interest in using a mobile app to communicate with their health care team.

In this sample of older patients with AF who used the internet, 53% reported using Facebook. We found that the oldest patients (aged 75-84 years and ≥85 years) were less likely to use Facebook, similar to national trends in social media use more generally among older adults [12]. Although social media use has increased dramatically among US adults aged ≥65 years in the past decade—from 2% in 2008 to 34% in 2016 [12]—the use of social media is more common among younger cohorts of older adults. In 2016, 47% of older adults aged 65 to 69 years, 41% of those aged 70 to 74 years, 24% of those aged 75 to 79 years, and 17% of those aged ≥80 years reported using social media [12]. The prevalence of Facebook use observed among online patients in our study is similar to these national estimates, considering that online patients represent 70% of the total SAGE-AF cohort. We also found that women were more likely to use Facebook than men, which aligns with data from the Pew Research Center that found that among US adults of any age, 62% of men and 74% of women used Facebook [23].

We found that patients with depressive symptoms were more likely to use Facebook than patients who were not depressed. Although a recent meta-analysis found depressive symptoms to be associated with more frequent social media use [30], the average age among participants in included studies was 22 years, and much less is known about depressive symptoms and social media use among older adults. Another limitation of previous research exploring the relationship between depressive symptoms and social media use is the lack of clarity about the directionality of the association—it may be that negative social comparisons on online social networks result in worsening of mood or it may be that individuals who are feeling depressed seek social support and connection online. In a national study of middle-aged and older women with chronic health conditions, women with depression reported more frequently relying on the internet for help and support than women without depression [31], suggesting that support may motivate Facebook use among older adults with AF with elevated depressive symptoms. Future research could explore how older adults with AF or other chronic health conditions with depressive symptoms utilize Facebook.

We found that 4 in 10 older patients with AF who used the internet were interested in an online AF patient community and that patients with higher education, obesity, more frequent online health information seeking, and daily Facebook use were more likely to express interest in an online AF patient community. In a national study of women with chronic health conditions, only 4% of women aged ≥65 years reported participating in an online discussion group, yet 27% of them said they would be somewhat or very interested in an online course or discussion group and 96% felt that it would be very helpful to get emotional support from people with similar problems [31]. This study extends this research by surveying interest in digital disease support among a contemporary community-based cohort of older adults and provides insights specifically into the interests of patients with AF. Our results indicate that among older patients with AF, those who are already engaged in online activities—online health information seeking and engaging with others via social media—are more likely to be interested in an online patient community. A study of middle-aged and older cardiac rehabilitation patients in Australia found that greater use of Facebook might be a predictor of greater willingness to participate in online patient support communities [13], concordant with our finding that patients who used Facebook daily were more likely to express interest in an online patient community for AF. Although we do have not information on patients’ social media activities, it may be that those who use Facebook daily are doing so to participate in a Facebook group for patients with AF.

In unadjusted analyses, younger patients, those with symptoms of depression or anxiety, patients who were bothered by AF symptoms, and those with lower AF treatment satisfaction were more likely to report interest in an online AF patient community. However, none of these factors were significantly associated with interest in an online patient community after adjusting for other factors, suggesting that this variance was captured by these other variables, such as frequency of Facebook use and online health information seeking. Indeed, in this study, we found that patients with depressive symptoms were more likely to use Facebook, and in previous research, patients who reported difficulty accessing medical care [32] or who reported problems with care coordination or care that was not patient-centered [33] were more likely to engage in online health information seeking or other online activities related to their health.

Recent qualitative research suggests that patients participating in online patient communities for AF find information and support provided through these communities to be helpful [14]. Results suggest that patients with AF make sense of their condition through communicating with other patients with AF online [14]. Members of the AF patient community seek knowledge about living well with AF and use the online community as a medium to discuss their personal experiences and gather information about the risks and benefits of different treatments [14]. Patients also seek information related to medication management in online communities, including concerns about safety and efficacy, dietary restrictions, and side effects [15]. Recent AF management guidelines recommend shared decision making with AF patients [34,35], and online resources are valuable sources of information and support for patients wishing to participate more meaningfully in their AF care. Clinics and health care providers may wish to provide their patients a list of online resources for AF (as seen in [35]) and encourage patients to join an existing online support community for AF, such as the American Heart Association and StopAfib.org’s MyAFibExperience, StopAfib.org’s discussion forum, Atrial Fibrillation Support Forum Facebook group, or the Lone Afib Forum. Given the potential benefits of engaging with other patients with AF and health care providers, clinicians may want to consider barriers to participation among AF patients not already engaging in digital disease management activities when recommending follow-up and disease education plans.

A little more than half of older adults with AF who owned smartphones and/or tablet computers (ie, mobile users) were interested in using a mobile app to communicate with their health care team. Data used in this study were collected before clearance from the Federal Drug Administration for the use of the Apple Watch and Apple Health app for managing AF electrocardiograms (ECGs), and as it becomes more commonplace for patients to send app-collected data to their health care team, interest in using a secure mobile app to communicate with one’s health care team may increase. We found that patients with higher education, men, those who engaged in online health information seeking more often, and those with mobile apps related to health were more likely to express interest in patient-provider communication via a mobile app. Studies assessing the usability of health-related apps among older adults [10,20,36], including those for AF [10,20], have enrolled more men than women, supporting the finding that a higher proportion of men are interested in using apps to manage their chronic condition.

Similar to previous research [37], older patients with AF who already had health-related apps were the most likely to express interest in communicating with their health care team via a mobile app—76% of patients who used health-related apps reported interest in app-mediated patient-provider communication. For the 42% of older patients with AF who do not currently use health-related apps but would be interested in communicating with their health care team using a mobile app, training older adults in basic smartphone functionality may aid in learning how to use an app-based intervention [36]. Clinics or health systems could explore using a secure mobile app to connect patients and health care providers, either via a stand-alone app or by using an app to access secure messaging functions of a patient portal.

In unadjusted models, patients aged 75 to 84 years and those aged ≥85 years were less likely to report interest in using a mobile app to communicate with their health care team. However, this age difference was no longer statistically significant after adjustment for the other factors examined, perhaps older adults were less likely to engage in online health information seeking, which was strongly associated with interest in app-mediated patient-provider communication. A study using data from the California Health Interview Survey found that compared with adults aged 60 to 74 years, those aged ≥75 years had 0.37 times the odds of engaging in online health information seeking [38], and in another study, patients in their 70s were less likely to use their health plan’s patient portal or send messages to their health care team through the platform [39]. Similarly, in unadjusted models, daily Facebook users were more likely to express interest in using an app than patients who did not use Facebook, but this difference was not significant in adjusted models, perhaps because of the overlap in patients who were high users of social media and those who had health-related apps or engaged in online health information seeking.

Although numerous apps related to the detection or management of AF exist, recent reviews have found that these apps vary in quality [40] and accuracy [21]. A recent review of 102 apps for patients with AF available on from Apple or Google Play found that the majority of the apps included information about AF and AF detection, and a quarter to a third of apps included symptom journals or medication reminders, and 1 app included a patient support community [40]. A quarter depended on an additional device [40]. The review did not report which apps included functionality allowing patients to communicate directly with their health care teams. Unfortunately, the review found that less than a fifth of apps (16% of apps from Apple and 13% from Google Play) included scientifically validated content [40]. Results of pilot studies of apps to help patients manage their AF appear promising [10]. In addition to including evidence-based AF information and behavioral strategies, apps to help patents with AF manage their health and communicate with their health care team should be developed to meet the user interface and functionality needs of older patients with AF [19,37].

### Strengths and Limitations

This study has additional strengths and limitations. The SAGE-AF cohort was contemporary and geographically diverse, and participants were enrolled from cardiology, primary care, and electrophysiology clinics, and the cohort focused on older patients who are often excluded from studies on technology. Although our sample had limited racial/ethnic diversity—91% of participants were non-Hispanic white—this is similar to the demographic composition of Medicare beneficiaries with incident AF (91% white) [41]. We did not collect information that would allow us to calculate patients’ financial resources relative to the federal poverty line, yet technology and social media are more common among adults with higher socioeconomic statuses [12,23]. The baseline interview did not include detailed questions about patients’ online activities, including what type of health information they sought online, participation in Facebook groups, and the use of specific health-related apps, and thus, we do not know whether patients used digital resources related to the management of their AF. We found frequent Facebook use, online health information seeking, and having health-related apps were related to interest in digital disease support; future research could explore associations between seeking information related to AF symptoms, treatment, or self-management or using health apps specifically related to AF and interest in digital disease support.

### Conclusions

A recent Cochrane systematic review of 11 trials concluded that the evidence was insufficient to infer that existing educational or behavioral interventions increased time in therapeutic range for patients with AF [8]. Given the complexity of medication adherence and other self-management activities between clinical encounters, digital health approaches may be an effective avenue for promoting adherence to medication and other lifestyle recommendations, including daily physical activity. A recent review of mobile health (mHealth) approaches to AF care summarized the use of technology for ECG or rhythm monitoring, heart rate monitoring, recording patient-reported symptoms and environmental factors, and medication adherence [9]. They also note challenges in mHealth research, including the need to demonstrate cost-effectiveness, increased workload for engaging with patients online, and reimbursement models for such care [9]. Although future research and policy work are needed to overcome these challenges, findings from this study indicate that many older patients with AF are interested in participating in an online patient community for AF and communicating with their health care team via a secure mobile app. Future research should explore these modalities for providing care to older patients with AF and supporting patients with their self-management activities, including symptom tracking and medication management.

In summary, we found that among patients aged ≥65 years with AF, 53% used Facebook, 40% were interested in an online AF patient support community, and 52% of mobile users were interested in using a mobile app to communicate with their health care team. Patients already engaged in online activities were more likely to express interest in these digital disease support modalities. However, even among the subgroup with the lower rate of expressed interest in these digital disease support modalities—patients aged ≥85 years—25% were interested in an online support community and 29% of mobile users were interested in using a mobile app to communicate with their health care team. Given the trends in technology adoption by generational cohorts [42], interest in digital disease support among older adults with AF is only likely to increase in the coming years. Additional research is needed on how to most effectively leverage social media and mobile apps to help older adults with AF manage their health. Understanding the characteristics of older online patients with AF who use social media and would be interested in digital tools to connect with other patients and communicate with their health care team can inform tailored behavioral interventions to help older patients with AF manage their health.

## Abbreviations

AF: atrial fibrillation
aOR: adjusted odds ratio
BMI: body mass index
ECG: electrocardiogram
mHealth: mobile health
NIH: National Institutes of Health
OR: odds ratio
SAGE-AF: Systematic Assessment of Geriatric Elements in Atrial Fibrillation
